# DCE and DSC perfusion MRI diagnostic accuracy in the follow-up of primary and metastatic intra-axial brain tumors treated by radiosurgery with cyberknife

**DOI:** 10.1186/s13014-019-1271-7

**Published:** 2019-04-15

**Authors:** Rosa Morabito, Concetta Alafaci, Stefano Pergolizzi, Antonio Pontoriero, Giuseppe Iati’, Lilla Bonanno, Michele Gaeta, Francesco Maria Salpietro, Enricomaria Mormina, Marcello Longo, Francesca Granata

**Affiliations:** 1 IRCCS Centro Neurolesi “Bonino-Pulejo”, Messina, Italy; 2 Section of Neurosurgery, Department of Biomedical, Dental Science and Morphological and Functional Images, University of Messina, Messina, Italy; 3 Unit of Radiation Oncology, Department of Biomedical, Dental Science and Morphological and Functional Images, University of Messina, Messina, Italy; 4 Radiology Unit, Department of Biomedical, Dental Science and Morphological and Functional Images, University of Messina, Messina, Italy; 5 Section of Neurosurgery, Department of Human Pathology, University of Messina, Messina, Italy; 6 Department of Clinical and Experimental Medicine, University of Messina, Messina, Italy; 7 Neuroradiology Unit, Department of Biomedical, Dental Science and Morphological and Functional Images, University of Messina, Messina, Italy

**Keywords:** DSC perfusion MRI, DCE Perfusion MRI, Radiation necrosis, Tumor recurrence, SRS

## Abstract

**Background:**

The differential diagnosis between radiation necrosis, tumor recurrence and tumor progression is crucial for the evaluation of treatment response and treatment planning.

The appearance of treatment-induced tissue necrosis on conventional Magnetic Resonance Imaging (MRI) is similar to brain tumor recurrence and it could be difficult to differentiate the two entities on follow-up MRI examinations.

Dynamic Susceptibility Contrast-enhanced (DSC) and Dynamic Contrast-Enhanced (DCE) are MRI perfusion techniques that use an exogenous, intravascular, non-diffusible gadolinium-based contrast agent.

The aim of this study was to compare the diagnostic accuracy of DSC and DCE perfusion MRI in the differential diagnosis between radiation necrosis and tumor recurrence, in the follow-up of primary and metastatic intra-axial brain tumors after Stereotactic RadioSurgery (SRS) performed with CyberKnife.

**Methods:**

A total of 72 enhancing lesions (57 brain metastases and 15 primary brain tumors) were analyzed with DCE and DSC, by means of MRI acquisition performed by 1,5 Tesla MR scanner. The statistical relationship between the diagnosis of tumor recurrence or radiation necrosis, decided according to clinicoradiologically criteria, rCBV and Ktrans was evaluated by the point-biserial correlation coefficient respectively.

**Results:**

The statistical analysis showed a correlation between the diagnosis of radiation necrosis or recurrent tumor with Ktrans (rpb = 0.54, *p* < 0.001) and with rCBV (rpb = 0.37, *p* = 0.002).

The ROC analysis of rCBV values demonstrated a good classification ability in differentiating radiation necrosis from tumour recurrence as well as the Ktrans. The optimal cut-off value for rCBV was k = 1.23 with 0.88 of sensitivity and 0.75 of specificity while for Ktrans was k = 28.76 with 0.89 of sensitivity and 0.97 of specificity.

**Conclusions:**

MRI perfusion techniques, particularly DCE, help in the differential diagnosis by tumor recurrence and radiation necrosis during the follow-up after radiosurgery.

## Background

According to the World Health Organization (WHO), high-grade gliomas are the most malignant primary intra-axial brain tumors in adults [1]. Brain metastases are the second most common intra-axial tumors, frequently arising from lung cancer, breast cancer, melanoma and renal cell carcinoma [1].

The current treatment of primary brain tumors or brain metastases is based on a combination of surgery, radiation therapy (RT) and chemotherapy, related to tumor histology and location [1–6].

The biological and clinical adverse effects of radiation therapy can be classified in acute, early-delayed and late-delayed onset, based on the time of occurrence and clinical presentation [6].

Acute and early-delayed radiation effects occur within the first 3 months after radiation therapy. Typically they spontaneously resolve and are associated to the clinical symptoms of the increased intracranial pressure [6, 7]. Acute and Early-delayed radiation effects may appear on imaging as non-enhancing white matter T2 high signal intensity and/or contrast-enhancing lesions in close to the irradiated tumor site [6].

Late-delayed radiation effects occur from 3 months to years after radiation treatment and are often progressive. Radiation necrosis is a common effect, it has increased incidence in white matter and shows imaging similar to tumor recurrence with contrast enhancement, edema and mass effect [6, 7]. The combined and adjuvant use of chemotherapy (i.e. cisplatin and carboplatin, doxorubicin, methotrexate and temozolamide) has been shown to improve survival in patients with brain tumor but, on the other hand, to increase the risk of developing tissue necrosis [2, 6, 8–10]. After conventional RT, many factors such as total irradiation dose, size of the irradiated volume, a small number of fractions, length of survival and patient age (young patients) at the time of the irradiation may predispose to radiation necrosis [2, 11, 12]. In stereotactic RT, the specific risk factors are the volume irradiated with 12 Gy dose (over 8 cc), previous irradiation and male sex [2, 8, 12].

Conventional MRI has shown significant limitations to determine whether or not an enhancing lesion represents tumor recurrence or not, i.e. radiation necrosis [1, 4–6, 13].

The differential diagnosis between treatment necrosis and tumor recurrence is crucial for diagnosis and treatment planning [5, 6, 14, 15]. Biopsy is reliable reach to a correct diagnosis, but it carries considerable surgical risks [6].

The aim of this study was to compare the diagnostic accuracy of a well-known MRI perfusion technique as DSC, and a relatively new MRI perfusion technique as DCE, in the differential diagnosis between radiation necrosis and tumor recurrence, in the follow-up of primary and metastatic intra-axial brain tumors after Stereotactic RadioSurgery (SRS) performed with CyberKnife.

## Materials and methods

### Study population and RT protocol

We retrospectively evaluated 28 patients (18 female and 10 male, mean age 61 ± 12 years, range 44–82 years) who, underwent perfusion MRI brain examination at our Institution in a period of two years (Table.1).

**Table 1:** Clinical characteristics of sample

Patients N (male/female)	Patient age Mean (SD)	Total Lesion N	Metastasis N (%)		Primary brain tumor N (%)		Radiation necrosis N (%)	Tumor recurrence N (%)	Salvage treatment N	
28 (10/18)	61 (±12)	72	57 (79.17)		15 (20.83)		42 (58.33)	30 (41.67)	4 metastases	
			Radiation necrosis (%)	Tumor recurrence (%)	Radiation necrosis (%)	Tumor recurrence (%)			Surgery N	SRS retreatment N
			34 (59.6)	23 (40.4)	8 (53.3)	7 (46.7)			1	3

Legend: *N* number, *SRS* Stereotassic Radio Surgery, frequency = %;

The patients were selected on the basis of the following inclusion criteria:

- Primary brain tumor treated with surgical intervention and with fractionated stereotactic radiosurgery (FSRS) with CyberKnife.
- Brain metastases treated with stereotactic radiosurgery (SRS) with CyberKnife.

Perfusion MR imaging of metastatic or primary tumors not treated with SRS or treated by other radiation therapies were excluded.

Thus, the final cohort consisted of 8 patients with intra-axial primary brain tumor (6 glioblastomas, 1 oligoastrocytoma and 1 sarcoma) and 20 patients with one or more intra-axial brain metastases (lung carcinoma, breast carcinoma, larynx carcinoma, colon cancer, prostate carcinoma, neuroendocrine tumor and melanoma).

Primary intra-axial brain tumors group consisted of 15 neoplastic volumes treatments because in 7 patients we demonstrated a tumor progression; intra-axial metastases group consisted of a total of 57 brain metastases.

Therefore a total of 72 enhancing lesions (57 brain metastases and 15 primary brain tumors) were studied and treated.

54 enhancing lesions were studied with combined DCE and DSC perfusion MRI protocol, 11 enhancing lesions were studied with DSC perfusion MRI, in 7 enhancing lesions DCE perfusion MRI was performed.

In total, rCBV for 65 enhancing lesions and Ktrans for 61 enhancing lesions were calculated.

The intra-axial brain tumors were treated with FSRS performed with CyberKnife (mean dose of 24.4 Gy- SD 4.7) and all patients underwent a combined administration of Temozolamide.

Intra-axial brain metastases were treated with CyberKnife (mean dose of 20.28 Gy- SD 2.8). All patients underwent chemotherapy.

### MR imaging acquisition protocol

Brain MRI acquisition was performed with 1,5 Tesla whole-body MRI equipment (Ingenia, Philips Medical System, Best, the Netherlands), using a 16-element phased array sensitivity-encoding (SENSE) head coil.

Conventional basal MR imaging protocol included axial T2-weighted Fast Spin Echo (FSE), axial T1-weighted Spin Echo (SE), axial Diffusion Weighted Imaging (DWI), 3D-Fluid Attenuated Inversion Recovery (FLAIR) sequences.

- T2-weighetd FSE images were acquired with the following parameters:TR/TE 4434/100 ms; flip angle 90°; 4 mm section thickness.
- T1-weighted SE images were acquired, before and after contrast medium administration, with the following parameters: TR/TE 634/15 ms; 5 mm section of thickness.
- DWI images were acquired with the following parameters: TR/TE 2946/86 ms; b-values 0 and 1000 s/mm^2^; 5 mm section thickness.
- FLAIR-3D images were acquired with the following parameters: TR/TE 5200/297 ms, TI 1660 ms; 2 mm section thickness.

Contrast-enhanced MRI examination included: DCE-DSC perfusion acquisition and 3D-T1-weighted MPR-GE and axial SE T1 weighted.

A total volume of 0.15 mmol/kg of Gadobutrol (Gadovist 1.0; Bayer Shering Pharma, Berlin, Germany) was used to acquire both DCE and DSC perfusion imaging.

First 40% of contrast volume was injected at 2 mL/s using an electronic power injector via 18-gauge antecubital venous access, for DCE perfusion scan.

After an 8 min interval gap between DCE and DSC, the remaining 60% of contrast medium at 5 mL/s, for DSC perfusion scan, was injected.

DCE MR imaging was performed by using a Spin Echo (SE) T1-weighted (TR/TE 20/2.1 ms), serially acquired before and after administration of contrast enhancement.

DSC imaging was performed by using a T2 weighted gradient echo EPI sequence (TR/TE 1461/40; flip angle 75°).

-3D-T1-weighted MPR-GE images were acquired with the following parameters: TR/TE 25/4.6 ms; 2 mm section of thickness.

Some patients performed only DSC or DCE because of an inadequate venous access.

DSC and DCE acquired images were transferred to a commercially available perfusion image processing offline workstation (Intellispace Portal IX; Philips Medical System) and imaging processing was performed.

### DCE images post-processing

Voxelwise maps of tissue contrast concentration with time were calculated by using pre- and post-DCE T1 maps combined with the tissue signal intensity-time curve. A small ROI was drawn at the center of the sagittal sinus and the mean phase was measured as a function of time. The phase-time curve was converted to the gadolinium-time curve. The gadolinium-time curve was used for a voxel-by-voxel estimation of plasma volume obtained from the phase-derived vascular input function (Vp) and the volume transfer constant obtained from phase-derived vascular input function (K^trans^).

### DSC images post-processing

MRI signal intensity was converted to a T2 relaxation rate and T2*weighted signal intensity-time curves were obtained. The time-dependent signal intensity data were therefore converted to contrast-agent concentration. An automated algorithm selected the most suitable pixels for Arterial Input Function (AIF) in a manually defined region of interest (ROI) covering the middle cerebral artery. After AIF measurement, the deconvolution analysis, a mathematical step allowing to remove from the contrast-agent concentration the temporal spread contribution associated with the AIF, was performed and the CBV maps were generated.

### Image analysis

The image analysis was performed by two neuroradiologists with expertise of at least 8 years. Axial SE T1-weighted post-contrast images were coregistered to the parametric maps. A contrast section containing the maximum diameter of the enhancing lesion near the surgical cave or involving the brain metastases was selected, and a single ROI was drawn freehand around the area of highest perfusion values. Areas of hemorrhage, blood vessels, susceptibility artifacts and cystic or necrotic changes were excluded.

For DSC images, the ROI on the CBV colorimetric map was drawn. Normalized rCBV was calculated as the ratio of the CBV within the enhancing region to the CBV within contralateral white matter, which was judged as normal.

For DCE images, the ROI on the Ktrans colorimetric map was drawn.

The final diagnosis between tumor recurrence and radiation necrosis was decided on the basis of clinical and neuroradiological criteria [5]. The lesions were considered to be radiation necrosis when the enhancing lesions disappeared or decreased in size on subsequent MR examinations, or they were present but unchanged on serial follow-up MR examinations for 10 months accompanied by neurologic improvement. The enhancing lesions increasing progressively in size were considered tumor recurrence [5, 13].

Morphological MRI data were correlated to perfusion parameters.

### Statistical analysis

The statistical relationship between the diagnosis of tumor recurrence or radiation necrosis, decided according to the clinicoradiologically criteria previously exposed (dichotomous variable) and rCBV and Ktrans was evaluated by the point-biserialcorrelation coefficient respectively. Inter-rater agreement was estimated through intraclass correlation coefficient (ICC) values to evaluate the agreement of rCBV and Ktrans measures considering the two raters. The maximum ICC value was 1.00 and it represented the stronger reliability. In general, ICC values above 0.75 indicated a good agreement.

Finally, ROC analysis was performed to calculate the diagnostic accuracy (Area Under the ROC curve, AUC) with an appropriate threshold. We compared rCBV and Ktrans according to the diagnosis of radionecrosis or tumor recurrence, performed by the clinicoradiologically criteria previously exposed (golden test). Sensitivity and specificity at designated threshold levels with their 95% confidence interval (CI) were evaluated.

Analyses were performed using an open source R3.0 software package (R Foundation for Statistical Computer, Vienna, Austria). A 95% of confidence level was set with a 5% alpha error. Statistical significance was set at *p* < 0.05.

## Results

### Point-biserial correlation coefficient and inter-rater agreement

A significant correlation between the diagnosis and rCBV (rpb = 0.37, *p* = 0.002), and a high significant correlation between the diagnosis and Ktrans (rpb = 0.54, *p* < 0.001) were found.

For inter rater excellent ICC values were detected in four comparison conditions, in which investigators alternated with each other. The resulting ICCs were high for the two comparison conditions: ICC1_rCVB_ = 0.74 (95% CI 0.05–0.93), and ICC2_Ktrans_ = 0.98 (95% CI 0.89–0.99).

### Diagnostic accuracy

**Fig. 1:**
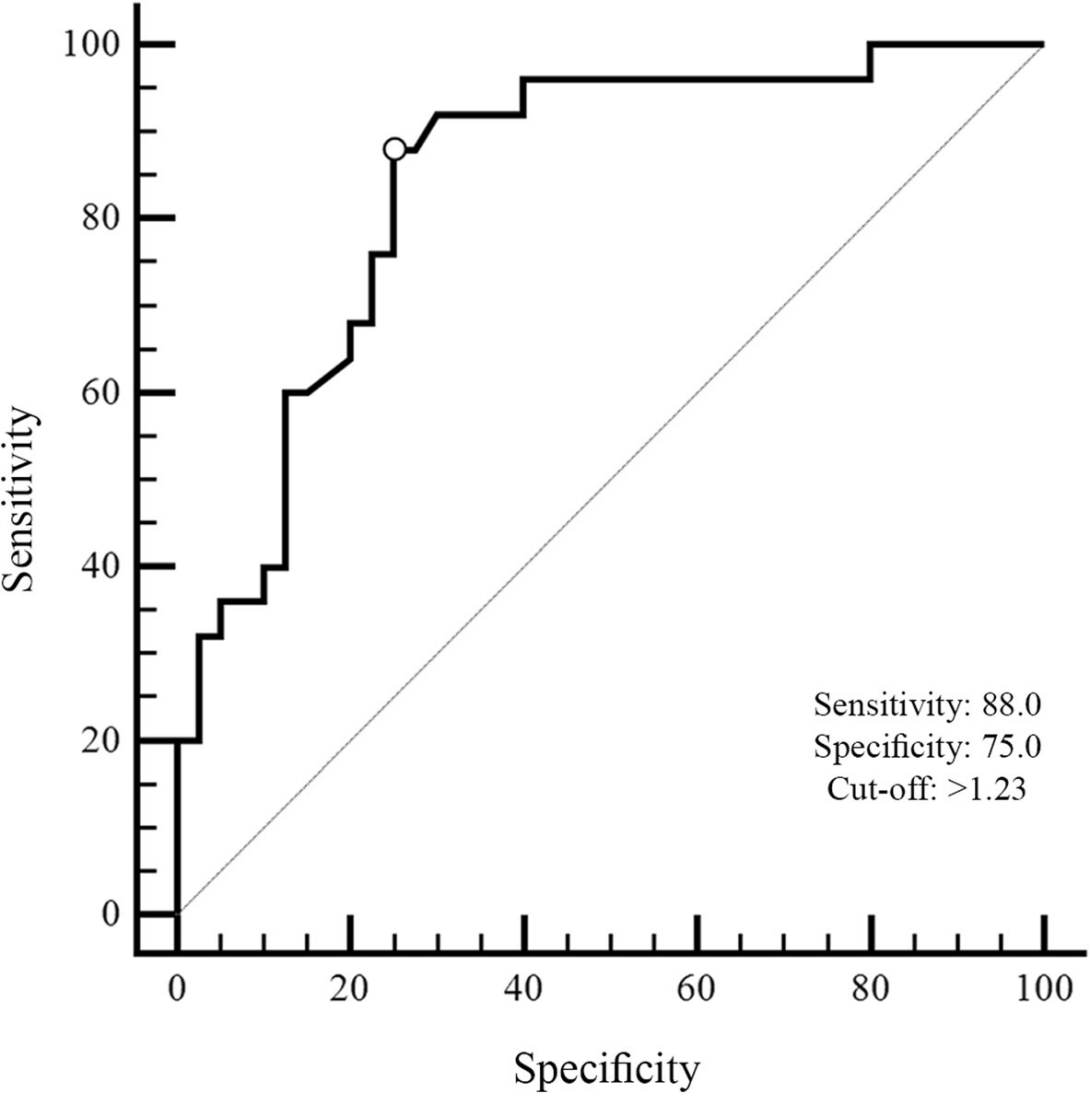
The ROC curve of rCBV on total scores of radiation necrosis and tumor recurrence groups. The value of the classification result produced best performance with an AUC value of 0.84

Receiver operating characteristic analysis was performed to measure the classification ability of the total score in radionecrosis and tumour recurrence for rCBV. The area under the curve (AUC) for the total score was the highest (AUC = 0.84, 95% CI = 0.72–0.92) indicating a greater classification ability in differentiating radiation necrosis from tumour recurrence (Fig.1). Threshold scores for each index were calculated by combining effects of sensitivity and specificity for each measure. The optimal cut-off value was k = 1.23 with 0.88 (95% CI = 0.69–0.97) sensitivity and 0.75 (95% CI = 0.59–0.87) specificity.

**Fig. 2:**
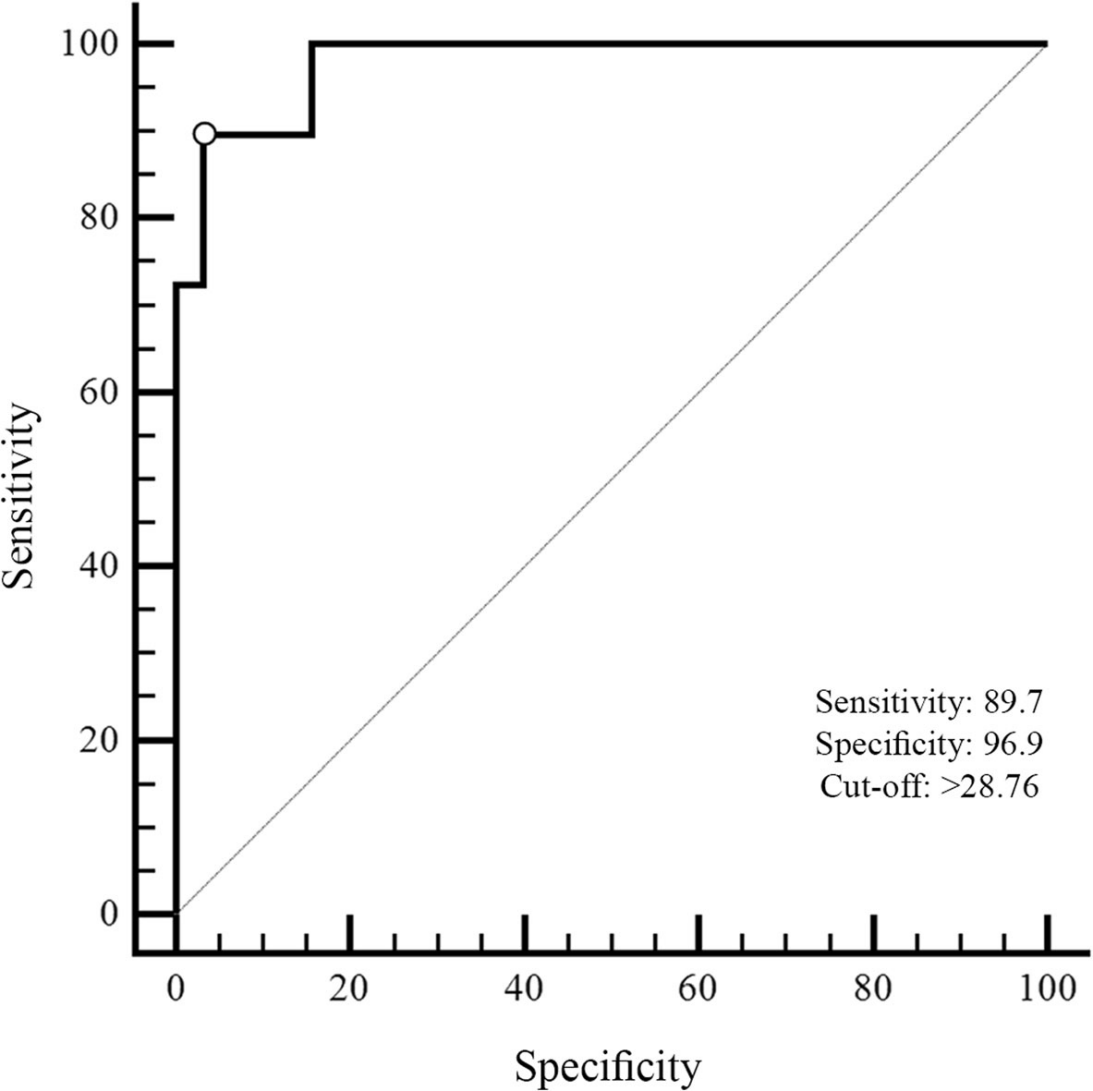
The ROC curve of Ktrans on total scores of radiation necrosis and tumor recurrence groups. The value of the classification result produced best performance with an AUC value of 0.98

The area under the curve (AUC) for the total score was the highest (AUC = 0.98, 95% CI = 0.90–0.99) indicating a greater classification ability in differentiating radiation necrosis from tumour recurrence for Ktrans (Fig.2).

Threshold scores for each index were calculated by combining effects of sensitivity and specificity for each measure. The optimal cut-off value was k = 28.76 with 0.89 (95% CI = 0.73–0.98) sensitivity and 0.97 (95% CI = 0.84–0.99) specificity.

**Fig. 3:**
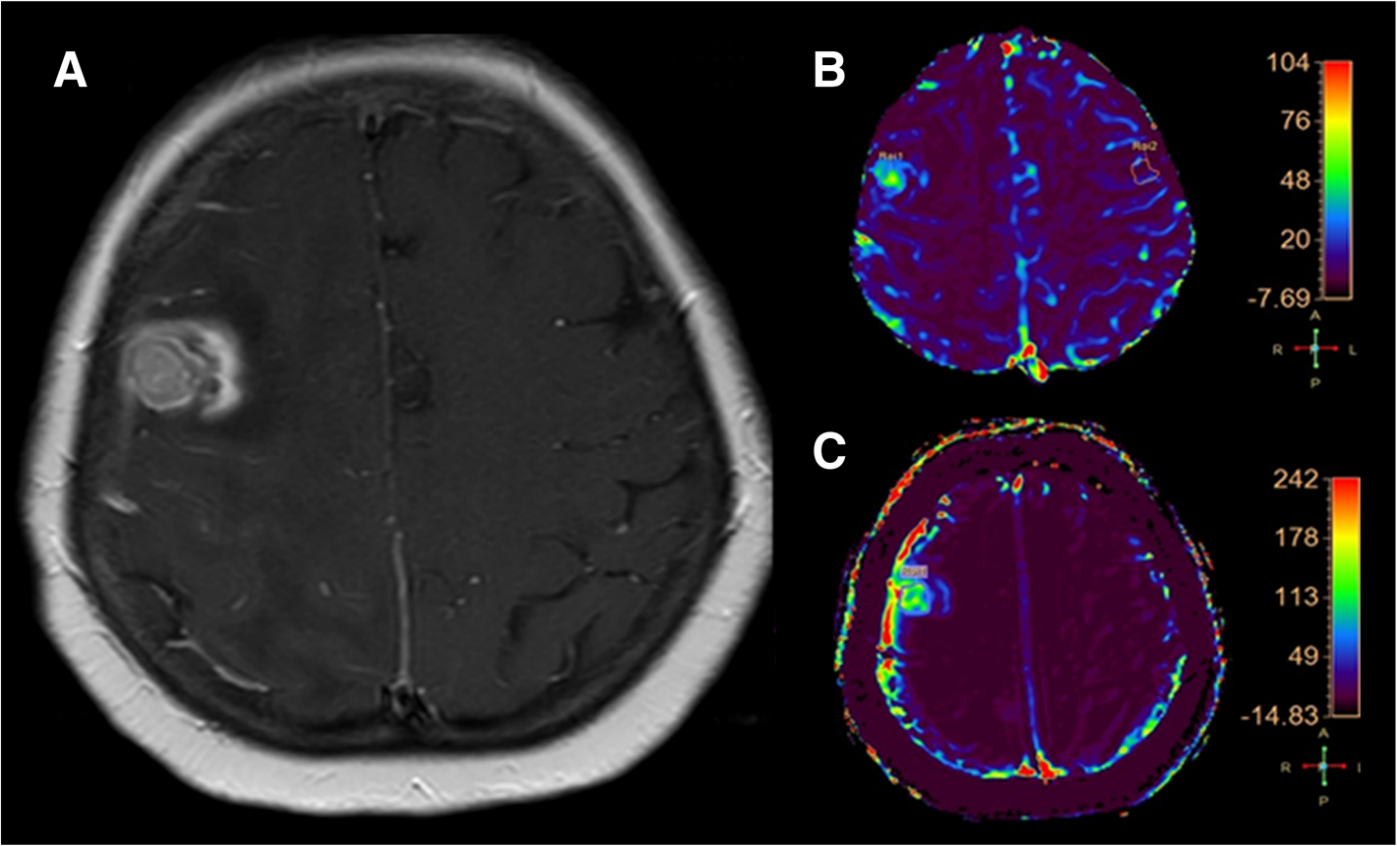
**a** (axial SE T1-weighted after Gadolinium administration), **b** (rCBV colorimetric map), **c** (Ktrans colorimetric map). Right frontal breast metastasis treated by SRS follow-up MRI examination. The enhancing lesion shows a central component with high rCBV and Ktrans values according to tumor recurrence and a peripheral component with low rCBV and Krans values according to the coexistence of radiation necrosis

Applying the cut-off, obtained with the ROC analysis, for Ktrans (28.76), 33 enhancing lesions were identified as tumor recurrence (Figs.1 and 2) and 28 as radiation necrosis (Fig.3).

**Fig. 4:**
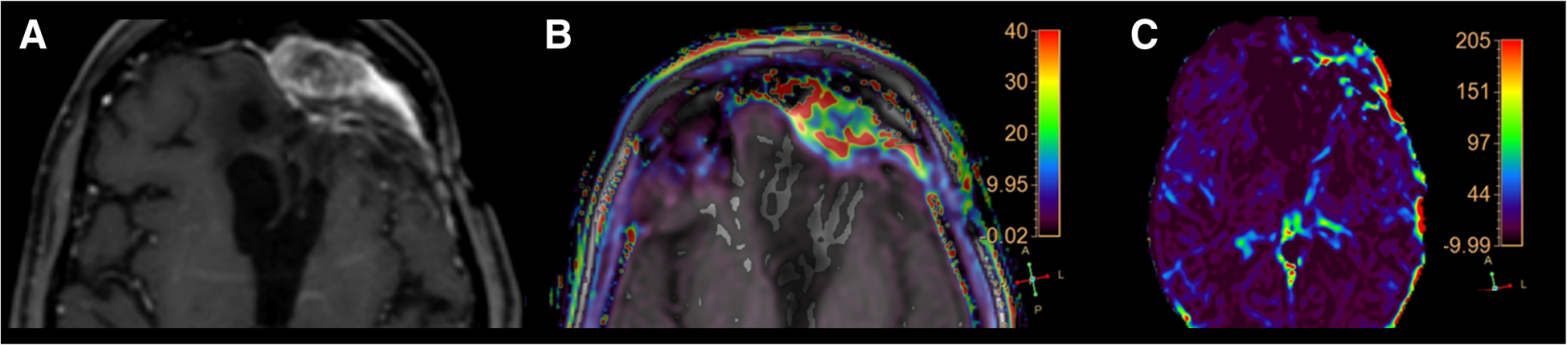
**a** (axial SE T1-weighted after Gadolinium administration), **b** (rCBV colorimetric map), **c** (Ktrans colorimetric map). Left frontal primary brain tumor (glioblastoma) treated by surgery and SRS follow-up MRI examination. The enhancing lesion shows high rCBV and Ktrans values, according to tumor recurrence

**Fig. 5:**
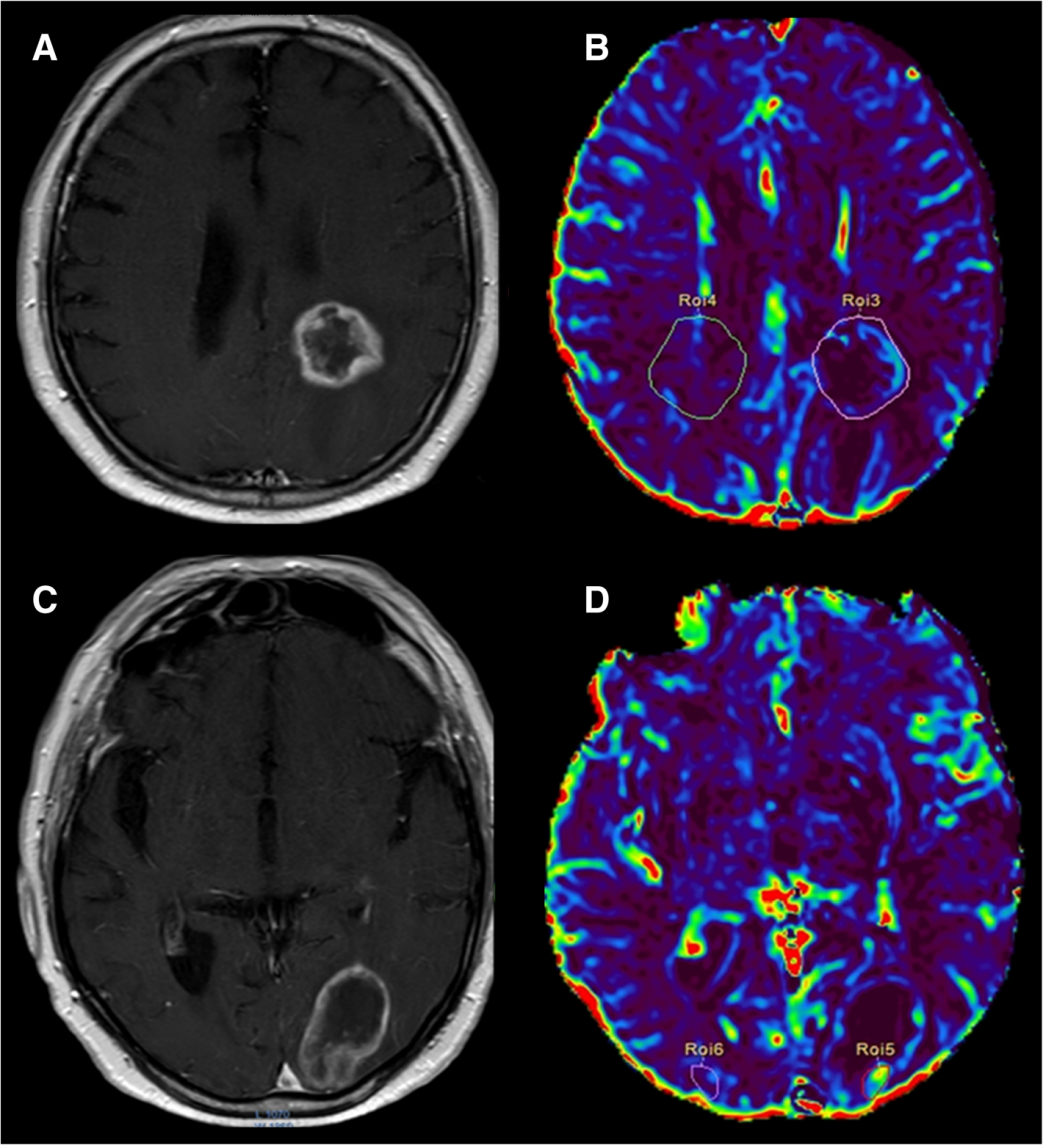
**a**-**c** (axial SE T1-weighted after Gadolinium administration), **b**-**d** (rCBV colorimetric map). Left paratrigonal and occipital brain metastases from lung cancer treated by SRS follow-up MRI examination. The enhancing left paratrigonal lesion shows low rCBV value according to radiation necrosis. The enhancing left occipital lesion shows a peripheral component with high rCBV value, according to tumor recurrence

Applying the cut-off, obtained with the ROC analysis, for rCBV (1.23), 33 enhancing lesions were identified as tumor recurrence (Figs.3 and 4) and 32 as radiation necrosis (Fig.5).

According to these results, 3 patients with MRI evidence of tumor recurrence underwent SRS re-treatment, 1 patient underwent surgery. The other patients with a diagnosis of tumor recurrence, were considered intractable for clinical general conditions and for an overall bad prognosis.

## Discussion

The differential diagnosis between radiation necrosis and tumor recurrence and/or progression is crucial for the evaluation of treatment planning and response in patients with primary or secondary brain lesions [2, 6].

The role of perfusion MR techniques in distinguishing recurrent tumor and radiation necrosis has been extensively studied [3–5, 7, 10, 13, 14, 16–18].

Dynamic Susceptibility Contrast-enhanced (DSC) and Dynamic Contrast-Enhanced (DCE) are the MRI perfusion techniques that use an exogenous, intravascular, no diffusible Gadolinium-based contrast agent. DSC emphasizes the susceptibility effects of Gadolinium-based contrast agents on the signal echo, using a series of T2- or T2*-weighted images. DCE exploits the relaxivity effect of Gadolinium-based contrast agent on the signal echo, acquiring serial T1-weighted images before, during and after its administration [1, 19–21].

Perfusion MR imaging is useful in differentiating highly vascularized recurrent tumor, characterized by an increase of perfusion parameters, from avascular necrosis [4, 5]. DCE is a relatively new perfusion MRI technique, that investigates microvascular structure and function by tracking the pharmacokinetics of the injected contrast medium as it passes through the tumor vasculature [22]. The increased tumor signal intensity in DCE MRI reflects its perfusion, vascular permeability and extracellular volume. The volume transfer coefficient of contrast between the blood plasma and the extracellular extravascular space (Ktrans) represents the permeability of the tumor vasculature and has been shown to be higher in tumor recurrence than in radiation necrosis [6, 16, 17, 22–26].

In our study, we performed follow-up MRI examination of 72 enhancing brain tumors, treated by SRS with CyberKnife technology, by using DCE and DSC perfusion MRI. We identified, according to clinical and neuroradiological criteria previously exposed, 43 radiation necrosis and 29 tumor recurrence or progression.

A significant statistical correlation between the diagnosis of radiation necrosis or recurrent tumor and the two perfusion parameters was evaluated. Particularly, Ktrans demonstrated a better correlation (rpb = 0.54, *p* < 0.001) compared to rCBV (rpb = 0.37, *p* = 0.002).

Moreover, a very good agreement between the two raters with excellent ICC values was demonstrated. ROC analysis showed a good classification ability in differentiating radiation necrosis from tumour recurrence for both rCBV (AUC = 0.84, 95% CI = 0.72–0.92) and Ktrans (AUC = 0.98, 95% CI = 0.90–0.99).

We were able to identify an optimal cut-off value for rCBV (k = 1.23 with 0.88 of sensitivity and 0.75 of specificity) and for Ktrans (k = 28.76 with 0.89 of sensitivity and 0.97 of specificity).

In our patients DSC and DCE perfusion MRI techniques showed a good diagnostic accuracy in the differential diagnosis between radiation necrosis and tumor recurrence. Particularly, Ktrans value with a 98% of sensibility and 97% of specificity demonstrated a better diagnostic accuracy compared to rCBV values that showed a sensitivity of 88% and a specificity of 75% respectively.

Our data seem to agree with previous DSC MRI perfusion studies. Sugahara et al. [5] demonstrated that enhancing lesions with a normalized rCBV ratio above 2.6 is considered tumor recurrence, while a normalized rCBV ratio less than 0.6 can be expression of radiation necrosis. They found a sensitivity of 50% and a specificity of 90% for perfusion-sensitive contrast-enhanced MR imaging; the cut-off value of the normalized rCBV ratios was 1.0. Barajas et al. [13, 27] demonstrated that perfusion MRI may be used to differentiate recurrent intra-axial metastatic tumor from radiation necrosis, using a cut-off rCBV value of 1.52 [13], and recurrent glioblastoma from radiation necrosis, applying a cut-off rCBV value of 1.75 [27]. Hu et al. [28] reported that radiation necrosis showed rCBV values from 0.21 to 0.71, whereas recurrent tumors have a rCBV values from 0.55 to 4.64.

Few papers in the litterature investigate the role of DCE perfusion MRI in the differential diagnosis between tumor recurrence and radiation necrosis and no specific cut-off emerged in Ktrans values.

Bisdas et al. [24] showed 100% sensitivity and 83% specificity for detecting the recurrent gliomas, using Ktrans cut-off value higher than 0.19.

Thomas et al. [16] used DCE MR imaging to differentiate pseudoprogression from recurrent glioblastoma, in patients treated with radiation and temozolamide after surgical resection. They identify a Ktrans (mean) thereshold of 3.5 for pseudoprogression and of 7.4 for tumor recurrence.

There are few reports in the literature which report combined DSC and DCE MR perfusion protocol [19, 20, 29], by calculating both rCBV and Ktrans. Particularly, Nguyen et al. [20] conclude that Ktrans and rCBV have a similar diagnostic accuracy in the preoperative evaluation of gliomas grading.

In our study, we used DSC and DCE perfusion MRI data to choose the most appropriate management of Cyberknife treated brain tumors. Particularly, 4 patients with MRI evidence of tumor recurrence or progression underwent surgery or RT re-treatment; patients with MRI evidence of radiation necrosis were addressed to a conservative treatment.

## Conclusion

In our experience, perfusion MRI techniques were highly diagnostic in discriminating tumor recurrence from radiation necrosis.

In particular DCE perfusion technique, with Ktrans value, demonstrated to be greatly useful in the clinical management of patients with RT treated brain lesions.

Therefore, these MRI techniques, although traditionally considered time-consuming, should be routinely performed in MRI follow-up, considering their high diagnostic value, especially in the neuro-oncology.
